# Supplementary material for: Transcriptional Controls for Early Bolting and Flowering in Angelica sinensis
Source: Plants (Basel). 2021 Sep 16;10(9):1931. doi: 10.3390/plants10091931 (PMC8468642; doi:10.3390/plants10091931)
Supplement: Supplementary file 1 [file plants-10-01931-s001.zip › Supplementary Materials-Plants.pdf]

Supplementary Materials

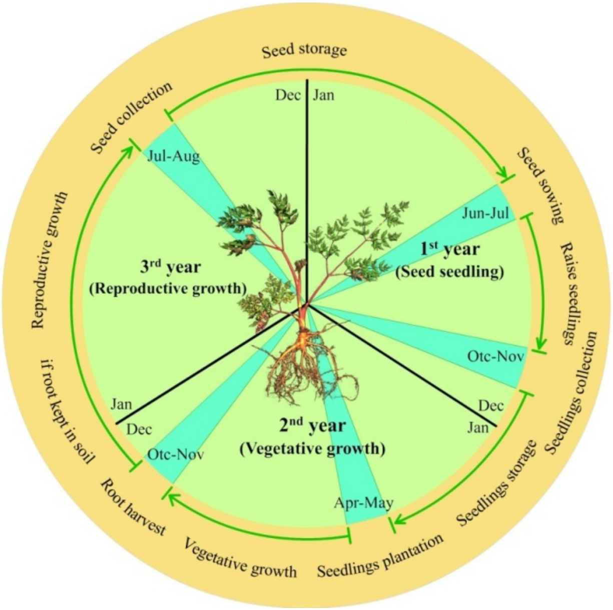

Figure S1: Growth and development of *Angelica sinensis* in a commercial production process.

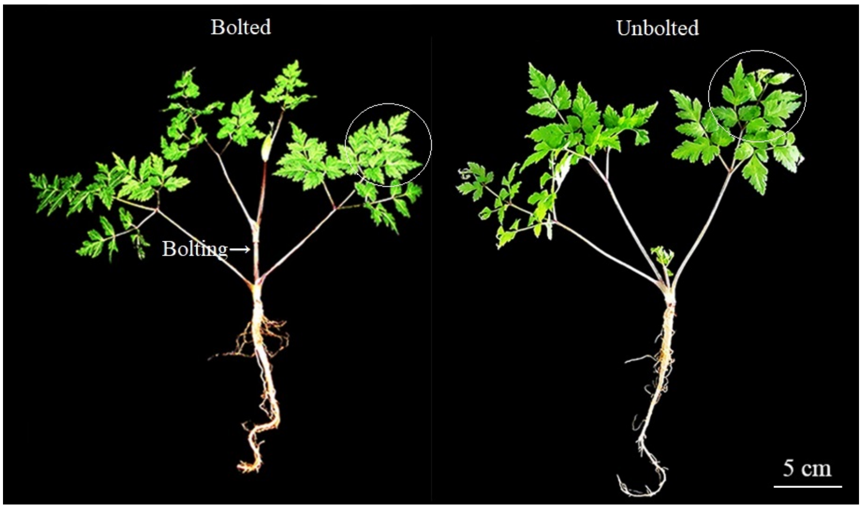

Figure S2: Two-year-old bolted and unbolted plants of *A. sinensis*. Bar represents 5 cm.

COL16, *COL2*, *COL3*, *HD3A*, *CO3*, *MIP1A* and *MIP1B*

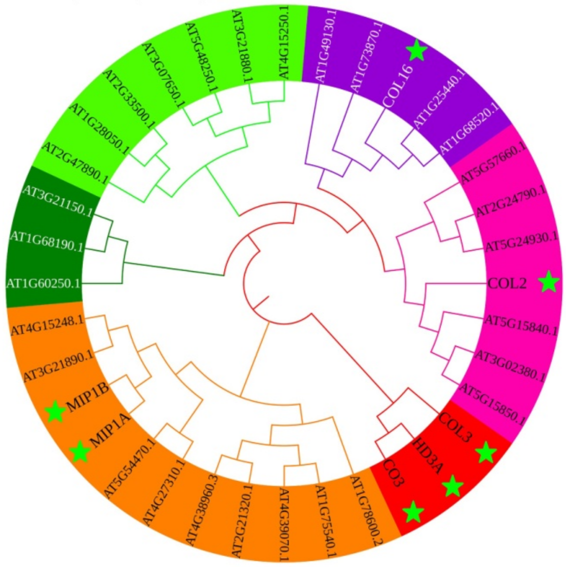

FTIP1

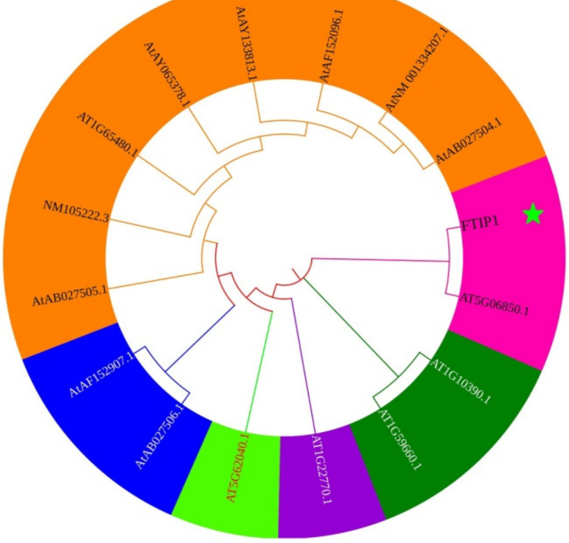

FD

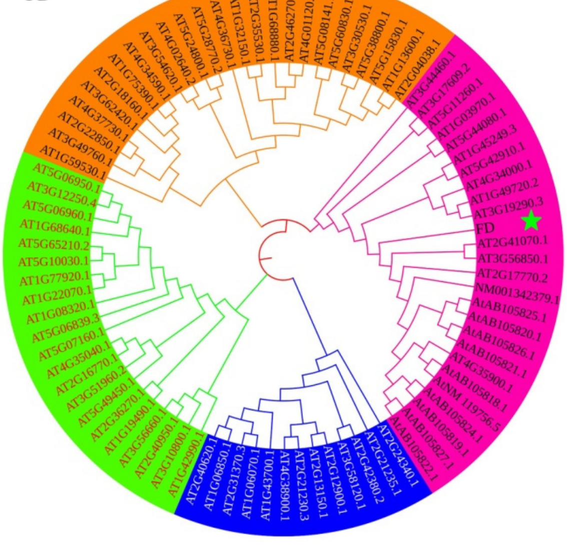

HDR1

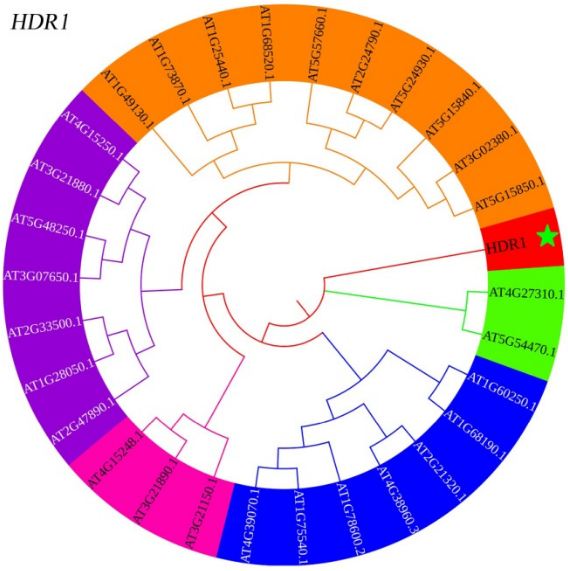

CDF2

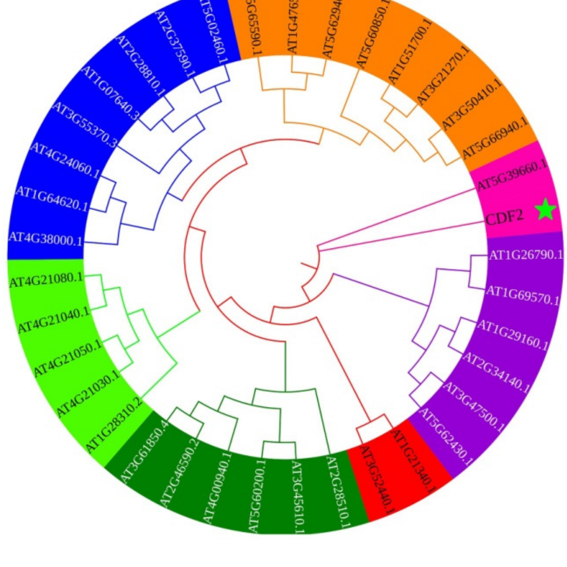

SVP, *AGL12*, *DEFA*, *SOC1*, *MADS8*, *AGL8* and *AP1*

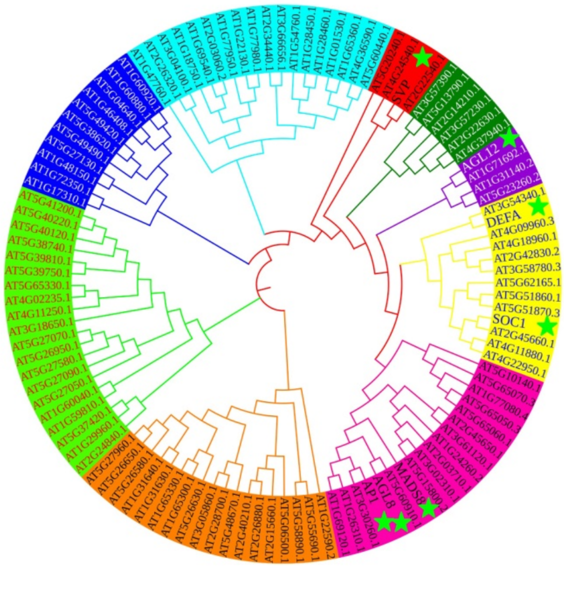

AS1 and *EFM*

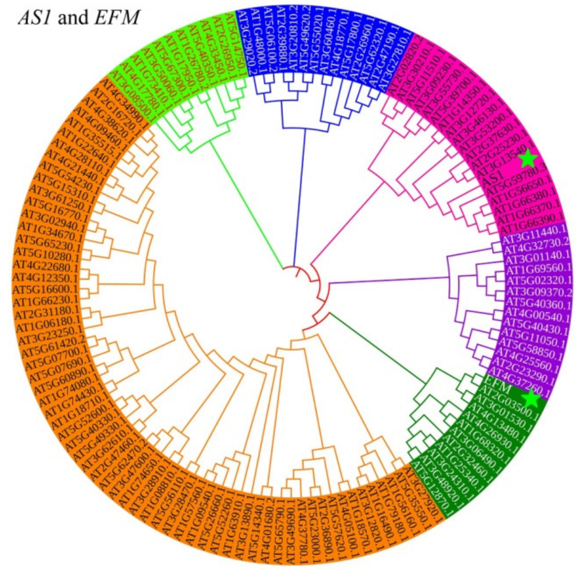

GA2OX6, *GA2OX8*, *GA20OX1*, *KO* and *GA2OX1*

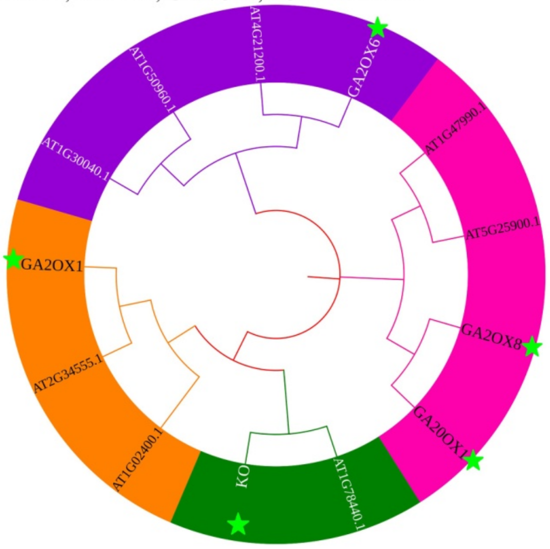

GAI and *GAIP*

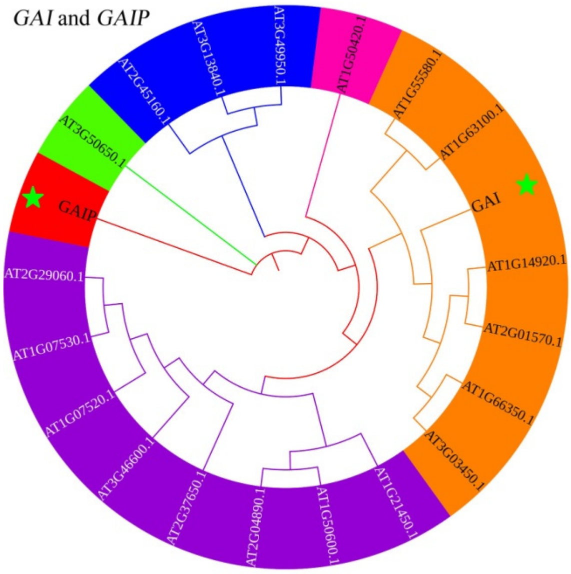

SUS7, *SUS1* and *SUS3*

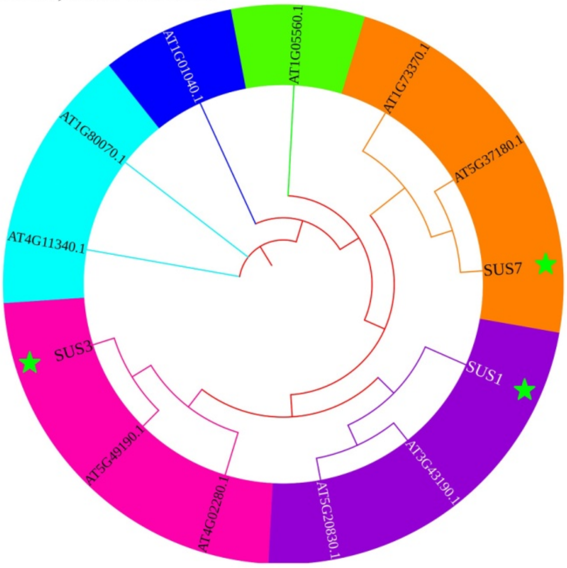

INVA, *INVE*, *INVB* and *INV Inh*

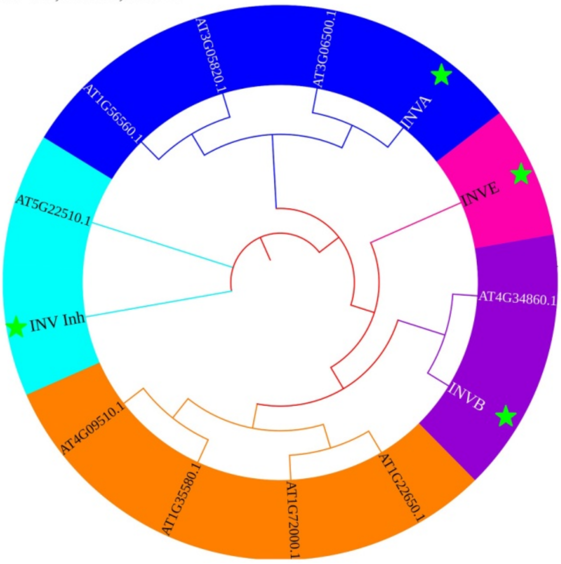

BAM1, *BAM3*, *BAM9* and *AMY1.1*

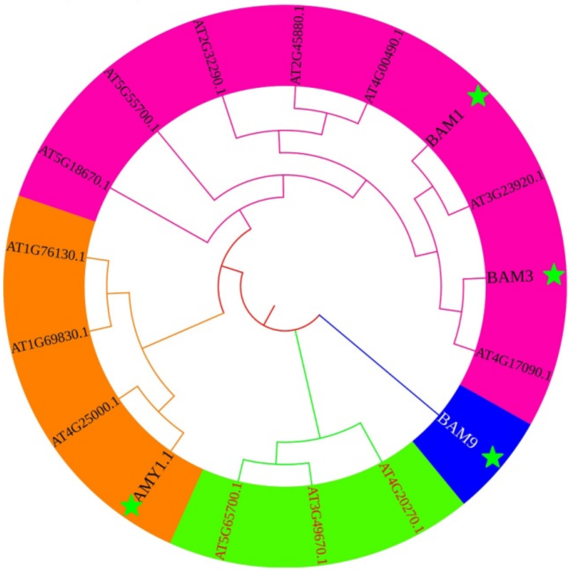

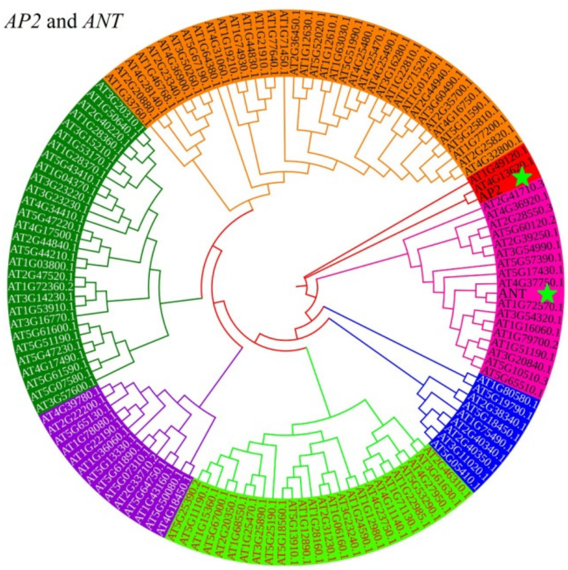

Figure S3: The cluster analysis of the 40 DEGs comparing with the families in *Arabidopsis thaliana*.

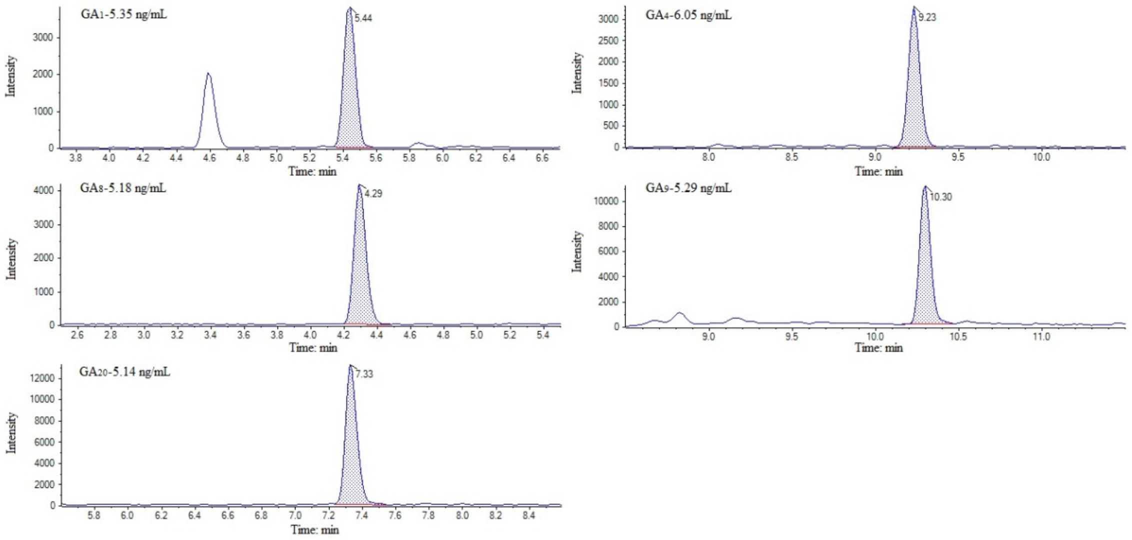

Figure S4: Representative chromatograms of reference standard of GA_1, GA_4, GA_8, GA_9 and GA_{20}.

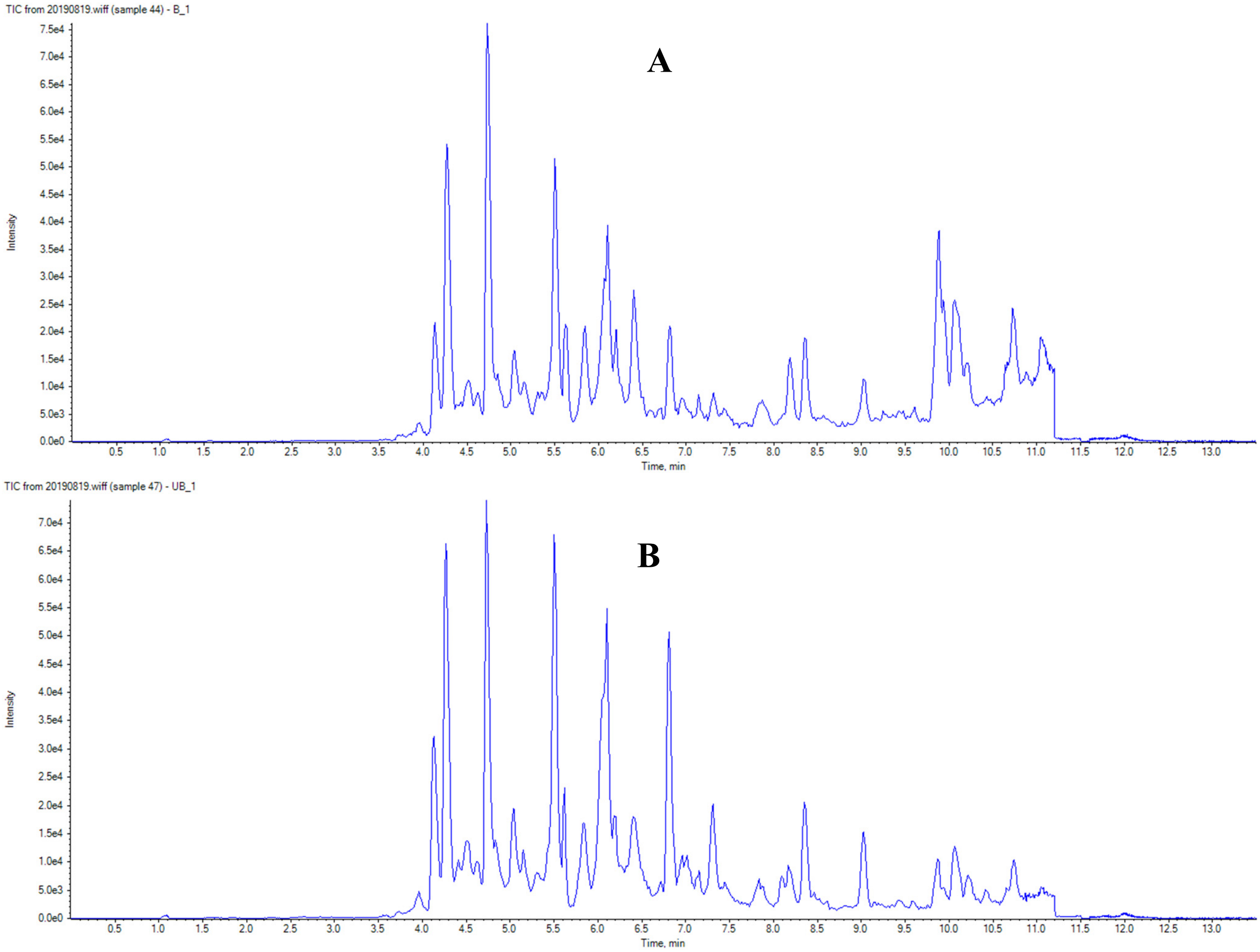

Figure S5: Representative chromatograms of bolted (A) and unbolted plants (B) of *A. sinensis*.

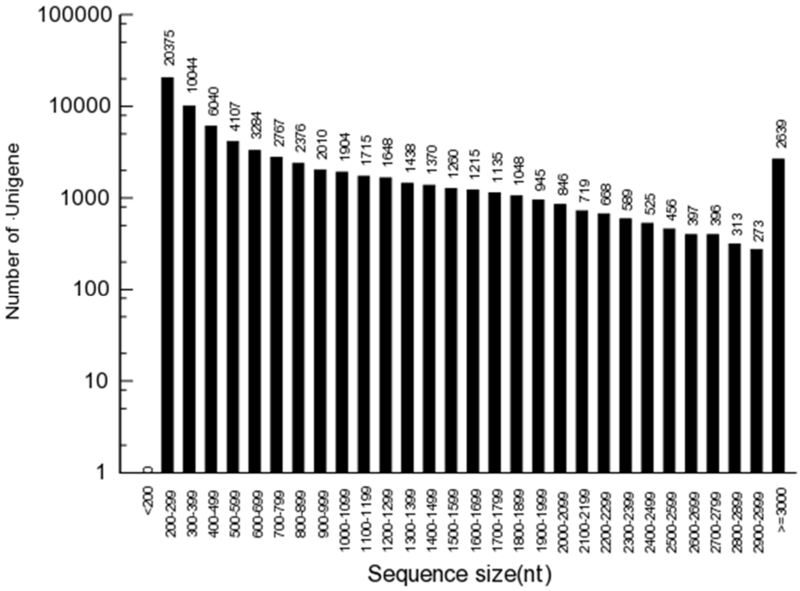

Figure S6: Length distribution of assembled unigenes.

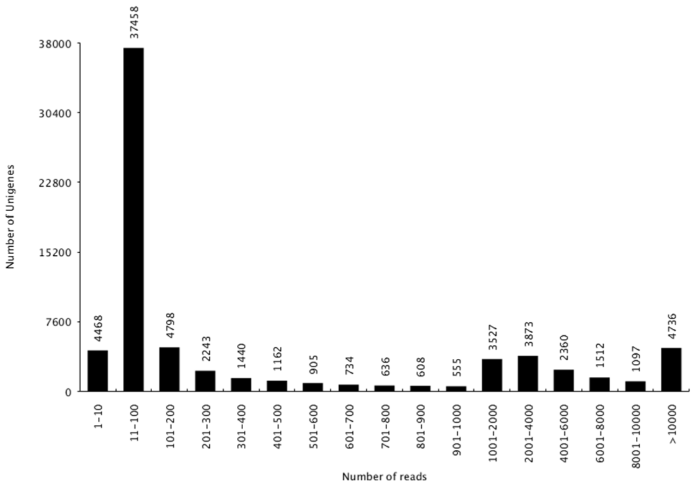

Figure S7: Read distribution of unigenes.

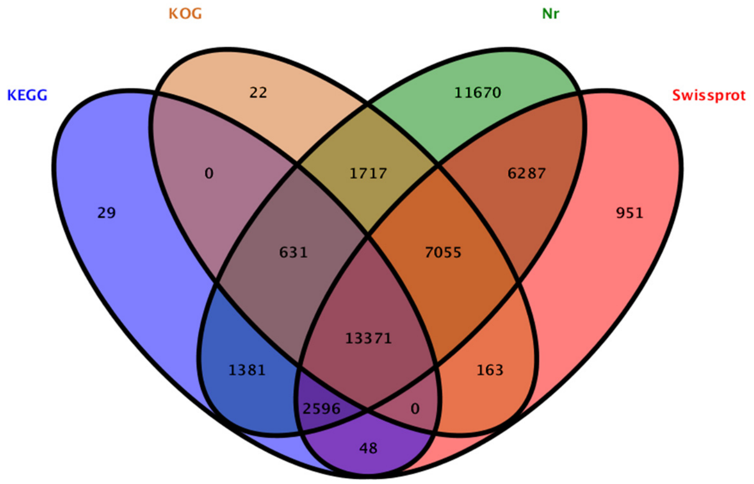

Figure S8: Basic annotation for all unigenes.

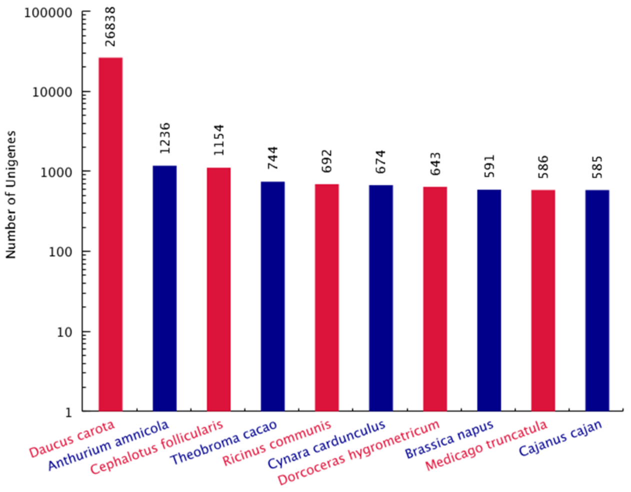

Figure S9: Top 10 plant species distribution of the total homologous sequences.

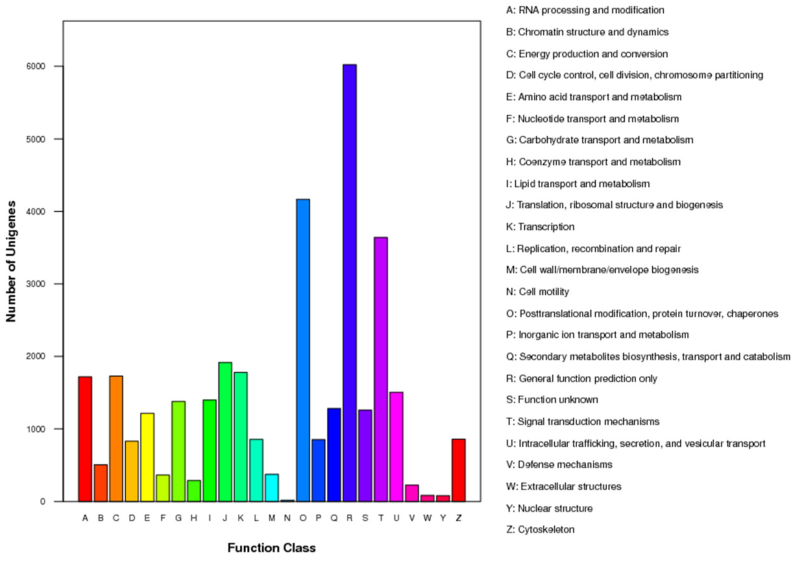

Figure S10: Distribution of unigenes in the transcriptome with KOG functional classification. Sequences have a KOG classification among 25 categories. Note: [A] RNA processing and modification; [B] Chromatin structure and dynamics; [C] Energy production and conversion; [D] Cell cycle control, cell division, chromosome partitioning; [E] Amino acid transport and metabolism; [F] Nucleotide transport and metabolism; [G] Carbohydrate transport and metabolism; [H] Coenzyme transport and metabolism; [I] Lipid transport and metabolism; [J] Translation, ribosomal structure and biogenesis; [K] Transcription; [L] Replication, recombination and repair; [M] Cell wall/membrane/envelope biogenesis; [N] Cell motility; [O] Posttranslational modification, protein turnover, chaperones; [P] Inorganic ion transport and metabolism; [Q] Secondary metabolites biosynthesis, transport and catabolism; [R] General function prediction only; [S] Function unknown; [T] Signal transduction mechanisms; [U] Intracellular trafficking, secretion, and vesicular transport; [V] Defense mechanisms; [W] Extracellular structures; [Y] Nuclear structure; [Z] Cytoskeleton.

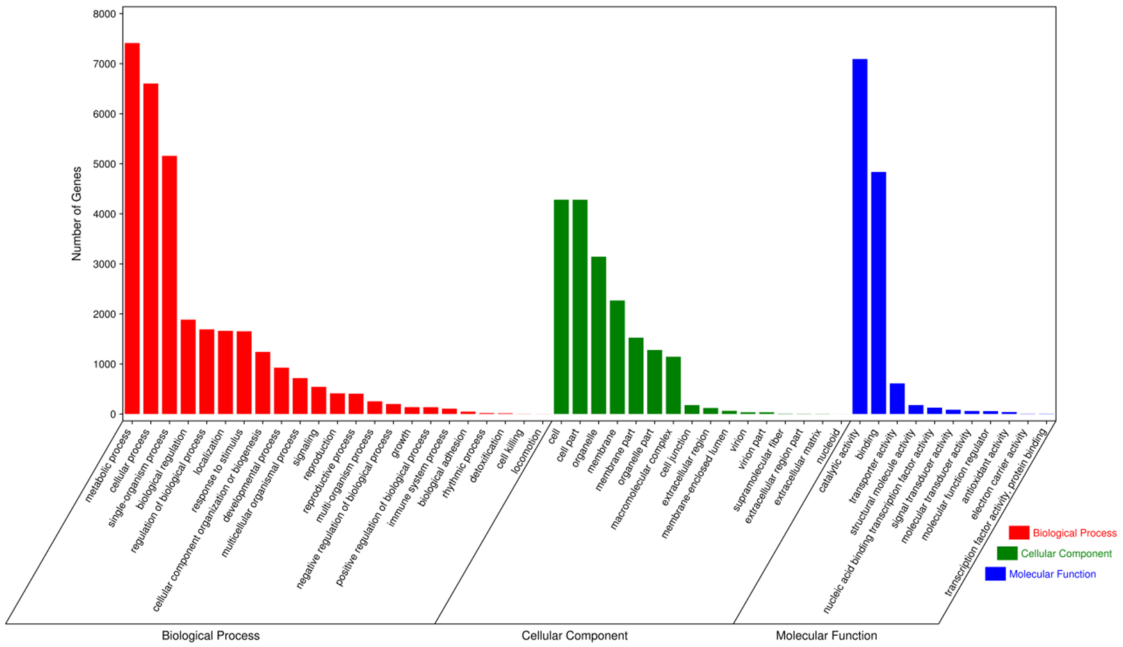

Figure S11: Functional classifications of GO terms of all assembled unigenes. Note: The results are summarized in three main categories: biological process, cellular component, and molecular function. The right y-axis indicates the number of genes in a category. The left y-axis indicates percentage of a specific category of genes.

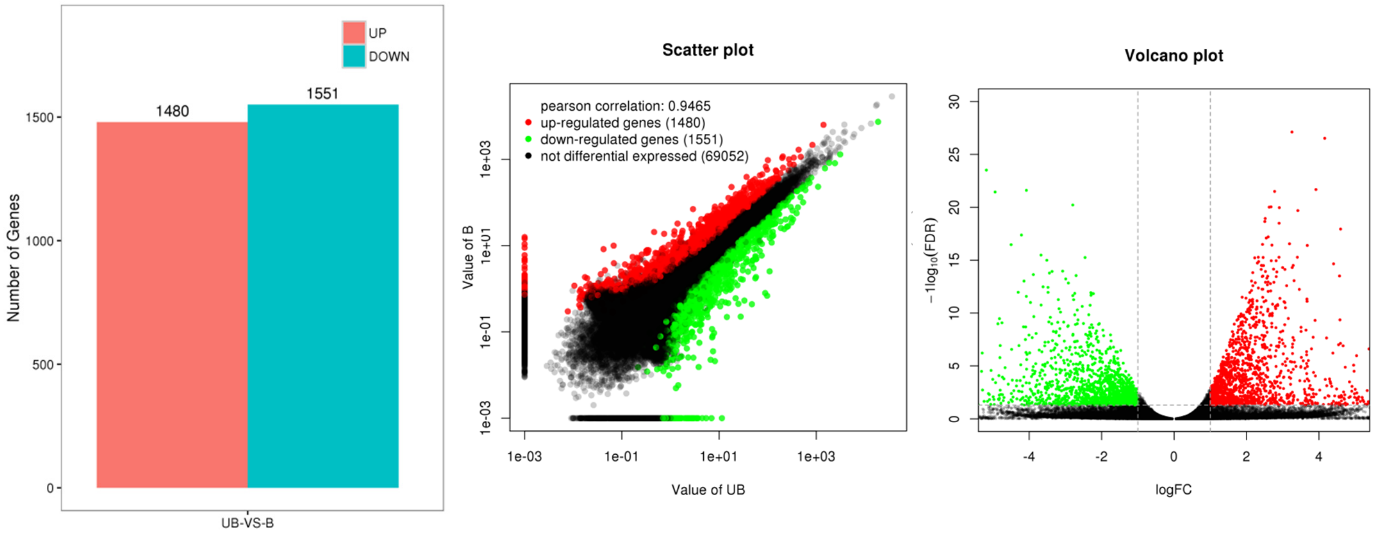

Figure S12: Number of DEGs, correlation and volcano plot for unbolted versus bolted plants. Note: A total of 2,645 DEGs were obtained after excluding 386 repeated genes from the originally generated 3,031 genes.

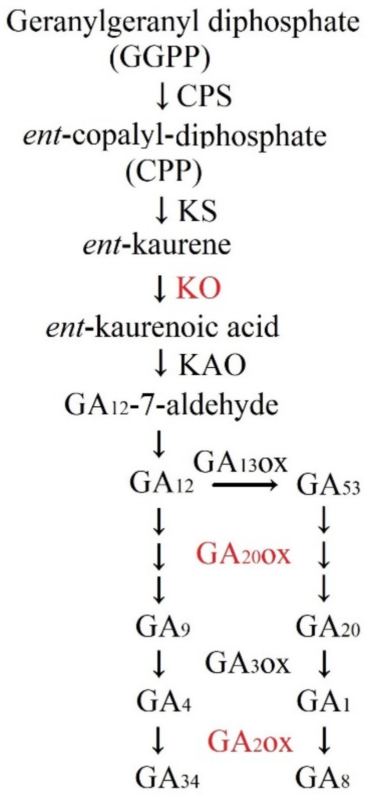

Figure S13: The GA biosynthesis pathways and the differentially expressed genes are highlighted in red.

Table S1: Sequences of primer used in the qRT-PCR analysis

Gene name	Sequences (5' to 3')	Amplicon size (bp)
ACT	Forward: TGGTATTGTGCTGGATTCTGGT<br>Reverse: TGAGATCACCACCAGCAAGG	109
**Floral development**		
SOC1	Forward: CGAAACGGCGAAATGGACTG<br>Reverse: CTGAATGTCTTGCCCAGCAG	200
MADS8	Forward: GAGATGAGCGGAGGCCAAAG<br>Reverse: AGCTTCCCCATGTTCTGTTCC	101
AGL8	Forward: CGGAGCAACAAAACCAAGAGAG<br>Reverse: CTCCGATGTTGCGTATGGCTT	102
AGL12	Forward: ATGGATGAACTGCATGTGCTTG<br>Reverse: ACCGGAACATCAATGTTACTAAACC	191
DEFA	Forward: ACTCCGGAATGAGATCGGC<br>Reverse: GCTCGCGTATGATGGCAAC	116
AP1	Forward: TCGAAGTCCTGCAACGGAAC<br>Reverse: CAGTTGGTTGTTCTGCCCC	200
AP2	Forward: CTCTTCGACAGCCACCTTCA<br>Reverse: AGGCTCCAACTGACTCGAAC	163
ANT	Forward: GCGAGGCGTAACAAAGACA<br>Reverse: CTGCTGTGGGAACTGATCTAGC	153
**Sucrose pathway**		
SUS1	Forward: ATGAAGTCCACACAGGAAGCC<br>Reverse: CGACGACAAGGTGATGAGTG	112
SUS3	Forward: GGAGAGAAGACTAACGTGCCT<br>Reverse: CGCGTAGCACTCTACTAGCC	169
SUS7	Forward: TGACTTTATCGTCATCGGTTGG<br>Reverse: AGAGCCGTGTTGAGTGTCT	166
INVA	Forward: TAAGTTGTGCGGTTTGCGAG<br>Reverse: ACTACACTCCTACCGCCAGT	174
INVB	Forward: TTGCCGTCCTTTTCACCTCC<br>Reverse: CTCATGCCCAAACTGTGACCT	132
INVE	Forward: CAATTCGGGAAATCGAGTCCT<br>Reverse: CTGCCGCACTAACACTTTCT	145
AMY1.1	Forward: AAATCCAGACACCGGCACTC<br>Reverse: ATCTCCAGCCAACGAATCCA	128
BAM1	Forward: GGGAAGCTCAGGTACCACTT<br>Reverse: GCACACATTTCCTCTGCTCC	127
BAM3	Forward: AGTCTCCGAGCAAGAAATTCG<br>Reverse: TGCTATGTGGCCGAGATGAC	127
BAM9	Forward: TTCCCGCAGAAAATCTGTTGA<br>Reverse: TGCGGAAATTGCTTTTGCGT	118
INV Inh	Forward: TCGAGCTTTATGATGATGCCCTC<br>Reverse: TAGCATTGTTCCTGGCGGTT	180
**GA pathway**		
KO	Forward: ACTTGCTGTGAGTGGCCCG<br>Reverse: AAGGAGGGAGATTCGAGGGTG	104
GA20OX1	Forward: AACCGTTCTACCAAGCGAAT	175

	Reverse: CCGGAGAGAAAGCCTCCTATG	
GA2OX1	Forward: ACCAACACATTGATTCCCCTC<br>Reverse: GGACATAGGTGAAGAGAAAAACTTG	171
GAI	Forward: GTATTCGGCTGGTCCACGTT<br>Reverse: AGCCAGCCACCTTACCAAAG	144
GA2OX6	Forward: GCCTTGACTTGCTAAGGGTG<br>Reverse: AGCCTCAGACCAAGAGAGTT	152
GA2OX8	Forward: AGTTGGCAGAAATCTTAGCGG<br>Reverse: CTCCGGTATTGGACATGGTGG	119
GAIP	Forward: TGTATCATCCCCCTCCTGCT<br>Reverse: GCATTCACTATCTCCTTTGCTTCC	132
**Photoperiodic induction**		
CO3	Forward: CTCAGCTTATGGGACCACC<br>Reverse: CGAGCACGTTGGCGAC	101
COL2	Forward: TTCATCCCCAGCAAGAGCAG<br>Reverse: GGATACAGAAACGCTGTGGC	131
HD3A	Forward: GAAAGTCCGAGGCCATCAGC<br>Reverse: CGGCAAAGTCTCTAGTATTGAATTG	121
FTIP1	Forward: AACGCTGACAAAATCACAAGCA<br>Reverse: GATTCCACTCGGGGTTCACT	193
FD	Forward: TACCACCCCCACCACTAAAC<br>Reverse: AGAGCTGCTGCTAGGGTTG	160
SVP	Forward: GCCGGATTAAGTCGCGTGAT<br>Reverse: TGGGATTAGCTTTCCACCTTTAGA	150
COL3	Forward: AAAGGTACAGAGCAAAGCGTG<br>Reverse: CCTTGAGCTTCATCATTGCGT	127
COL16	Forward: GATGGGTCAGCAGAGACGAT<br>Reverse: TCGCTGTTTGTCATTTCCGT	116
AS1	Forward: ATCCCGACCAACCTGCTTC<br>Reverse: AAGTTGGGAGTGGAGCTGTT	138
CDF2	Forward: TCGATCCCCAACAACGAAGAG<br>Reverse: ATGTTGTTGGACCACCCCTC	196
MIP1A	Forward: GCGATACAAGGGTTCATGCTG<br>Reverse: GAGTCCGATGAGATAGCGGTG	104
MIP1B	Forward: TGATTGCCACGCTCTAACCC<br>Reverse: GGCCTAATGAGTTGGTTGGTG	156
HDR1	Forward: GCAAGAGATCAGGACAGGGAG<br>Reverse: TGGTTTTCCAGCATTGTGTTTTG	146
EFM	Forward: TGACTTGCACCGGAGATTTGT<br>Reverse: TTTGAACTCGGGCCAACCA	200

Table S2: Calibration curves of GA_1, GA_4, GA_8, GA_9 and GA_{20}.

GAs	Calibration curves	Related coefficient
GA_1	y = 3549.48823 x + 133.18755	(r = 0.9923)
GA_4	y = 2527.09342 x + 574.86954	(r = 0.9905)
GA_8	y = 4004.18848 x + 320.37962	(r = 0.9934)
GA_9	y = 9565.89109 x + 339.64459	(r = 0.9949)
GA_{20}	y = 12108.82863 x + 1112.84346	(r = 0.9928)
